# A new protocol for renal collecting system sterilization with antibiotic irrigation during lithotripsy in retrograde intrarenal surgery: a prospective, comparative study

**DOI:** 10.1007/s00345-024-04903-8

**Published:** 2024-04-10

**Authors:** Ali Kaan Yildiz, Arif Bayraktar, Turgay Kacan, Demirhan Orsan Demir, Yusuf Gokkurt, Bugra Bilge Keseroglu, Tolga Karakan

**Affiliations:** 1 Department of Urology, Ankara City Hospital, Ankara, Turkey; 2 Department of Urology, Ankara Training and Research Hospital, Ankara, Turkey; 3 Department of Urology, University of Health Sciences (Sağlık Bilimleri Üniversitesi), Ankara City Hospital,, Ankara, Türkiye

**Keywords:** Kidney, Stone, Retrograde, Intrarenal, Infectious, Complication

## Abstract

**Purpose:**

To present a new protocol using antibiotic irrigation during lithotripsy in retrograde intrarenal surgery (RIRS) to provide sterility of the renal collecting system.

**Methods:**

This prospective study included 102 patients who underwent RIRS between January 2022 and August 2023. The patients were examined in two groups as those who received antibiotic irrigation (*n*:51) and standard irrigation (*n*:51). In the antibiotic irrigation group, 80 mg of gentamicin was dissolved in normal saline in a 3 L irrigation pouch to obtain a 26.7 mg/L concentration. In the standard irrigation group, normal saline was used. Preoperative information, including age, sex, body mass index (BMI), ASA score, stone side, volume, and density, and the Seoul National University Renal Stone Complexity (S-ReSC) score. The groups were compared with respect to postoperative fever (> 38 °C), urinary tract infection (UTI), systemic inflammatory response syndrome (SIRS), infectious complications such as sepsis, and stone-free rate.

**Results:**

No statistically significant difference was determined between the groups with respect to age, sex, BMI, ASA score, stone side, volume and density, and S-ReSC score (*p* > 0.05 for all). Statistically significant differences were determined between the groups with respect to postoperative fever (*p* = 0.05), SIRS (*p* = 0.05), and hospital length of stay (*p* = 0.05). Sepsis was observed in one patient in the standard irrigation group and in none of the antibiotic irrigation group.

**Conclusion:**

The reliability, efficacy, and utility of antibiotic irrigation during lithotripsy in RIRS were presented in this study as a new protocol for sterilization of the renal collecting system which will be able to reduce infectious complications.

## Introduction

The prevalence of urolithiasis is high and continues to increase [1]. With the rapid developments in urological endoscopy, minimally invasive surgery has become the ideal treatment method for kidney stones. Retrograde intrarenal surgery (RIRS) performed with flexible ureteroscope and laser lithotriptor is a safe and effective minimally invasive procedure for kidney stones. The European Association of Urology (EAU) guideline for the treatment of kidney stones recommends RIRS as the first option for stones < 2 cm [2].

In the endoscopic treatment of urinary system stones, postoperative infectious complications constitute 50% of all complications [3]. One of the most important factors in protection against infectious complications is a negative preoperative urine culture. However, even if preoperative urine culture is negative in patients undergoing RIRS, postoperative fever has been reported at the rate of 14.6% [4]. In the endourological treatment of kidney stones, stone and renal pelvis culture has been found to have higher efficacy in the prediction of infectious complications than mid-flow urine culture, and even if preoperative mid-flow urine culture is negative, the complication rates are increased if there is bacterial production in stone or renal pelvis culture [5, 6].

With the clear definition of the risk factors for infectious complications, it has been attempted to produce the solutions for sterilization of the urinary system. Many studies have been conducted related to preoperative prophylaxis in RIRS, and various results have emerged, but as yet there is no study related to sterilization of the renal collecting system.

The aim of this study was to provide collecting system sterilization during lithotripsy with the use of antibiotic irrigation solution in RIRS and to report the results related to the utility and reliability of an antibiotic irrigation protocol.

## Methods

This prospective, comparative study included 102 patients who underwent RIRS because of kidney stones between January 2022 and August 2023.

### Data and clinical diagnostic methods

The patients included in the study were those who were determined with kidney stone with no stone in the ureter. The primary outcome was the difference in the rates of postoperative fever between the two groups. The study exclusion criteria were defined as the presence of a stent or nephrostomy tube when starting the operation, serum creatinine value > 1.5 mg/dL, pathological ureter stricture, a history of radiotherapy, solitary kidney, or pregnancy.

The basic data recorded for each patient included age, gender, body mass index (BMI), ASA score, side, volume, density of the stone, and the Seoul National University Renal Stone Complexity (S-ReSC) score. Stone volume was calculated as the length in millimeters of the stone multiplied in three axes, and the total stone volume was defined as the cumulative stone volume. The preoperative evaluations of all the patients included X-ray imaging and non-contrast abdominal computed tomography (CT) scans.

### Surgical procedure

In all the cases, urine culture was taken 1 week before ureteroscopy and the urine culture was checked on the day before the operation. The final urine culture results of all the operated patients were sterile. Prophylactic antibiotics were administered preoperatively as an intravenous single dose of 2 g ceftriaxone depending on the kidney functions of the patient. In the antibiotic irrigation group, by dissolving 80 mg gentamicin in a 3 L irrigation pouch of normal saline, a 26.7 mg/L concentration was obtained. Following general anesthesia, the patients were placed in the lithotomy position, and low-pressure perfusion was obtained by placing 3 L irrigation solution 100 cm above the patient. A semi-rigid ureteroscope (6.5/8.5 F, Karl Storz, Tuttlingen, Germany) was advanced from the urethra to the bladder, and then, both ureter orifices were visualized. Under fluoroscopy guidance, a 0.035-inch soft-tipped guidewire was advanced from the ureteral orifice and reached the renal pelvis. In cases where the semi-rigid ureteroscope could not pass through the ureter, the operation was postponed by placing a double-J ureteral 4.8 Fr stent. Balloon or serial dilation was not performed in any patient where the ureter could not be passed. In the cases where the semi-rigid ureteroscope reached the pelvis, a ureter access sheath (internal diameter 9.5 F, external diameter 11.5 F, Cook Medical, Bloomington, USA) was placed over the guidewire and checked with fluoroscopy, and then, the procedure was started with a flexible ureteroscope (7.5 F, Karl Storz Flex-X2S, Tuttlingen, Germany). The ureteral access sheath could not be placed in six patients in the antibiotic irrigation group and seven patients in the standard irrigation group, and the flexible ureteroscope was placed directly over the guidewire in these patients. Stone fragmentation was performed using 273-micron holmium:yttrium–aluminum–garnet (Ho:YAG) laser. Laser lithotripsy was performed with dusting technique settings (0.3–0.5 J and 20 Hz). There was no fragment extraction during lithotripsy, and no extraction device such as a basket or grasper was used. At the end of the procedure, a 4.8 F double-J ureteral stent was placed to remain for 3 weeks postoperatively. At 3 weeks after the operation, the double-J stent was removed from all the patients, and the stone-free status was evaluated with non-contrast CT at 4 weeks postoperatively. Removal of the double-J stent was performed as an outpatient procedure for all the patients, and no patient required hospitalization for this. Stone-free status was defined as no residual stone or clinically insignificant residual stone of < 2 mm. All the surgical procedures were performed by two surgeons with experience of an average of 200 urinary system stone operations per year.

Postoperative fever was defined as body temperature > 38 °C within 48 h postoperatively. Urinary tract infection (UTI) was evaluated with urine and blood cultures. The criteria for systemic inflammatory response syndrome (SIRS) were defined as at least two of the following: hyperthermia (> 38 °C) or hypothermia (< 36 °C), respiratory rate > 20, tachycardia (> 90 bpm), and white blood cell (WBC) > 12,000 mm^3^, < 4000 mm^3^. Confirmed or suspected infection with SIRS was defined as sepsis.

All the patients were evaluated with respect to operating time, stone-free rate, postoperative fever, UTI, SIRS, sepsis, tachycardia, tachypnea, abnormal WBC count, admission to the surgical intensive care unit (SICU), change in creatinine on postoperative day 1, length of postoperative hospital stay, and rehospitalization within 30 days of RIRS.

All patients are hospitalized for at least one night of observation following RIRS. Therefore, the laboratory studies were made on the morning of the first postoperative day. The monitoring of vital signs was continued in the recovery room immediately after the operation and then on the ward.

### Statistical analysis

Continuous variables were stated as mean ± standard deviation values if the data conformed to a normal distribution and as median (interquartile range (IQR)) if the distribution was not normal. Categorical variables were stated as number (*n*) and percentage (%). In the comparisons of categorical data between the groups, the Pearson chi-square test and Fisher’s exact test were used. A value of *p* < 0.05 was accepted as statistically significant.

## Results

From the total of 131 patients identified at the start of the study, 11 did not meet the study inclusion criteria (8, previous double-J stent; 2, solitary kidney; and 1, high serum creatinine); 5 did not accept the operation; then of the 115 patients who started the study, the semi-rigid ureteroscope did not pass through the ureter in 9 patients; and 4 did not attend the follow-up examinations. Thus, the study was completed with a total of 102 patients. The groups comprised 51 patients in the antibiotic irrigation group and 51 in the standard irrigation group.

**Fig. 1:**
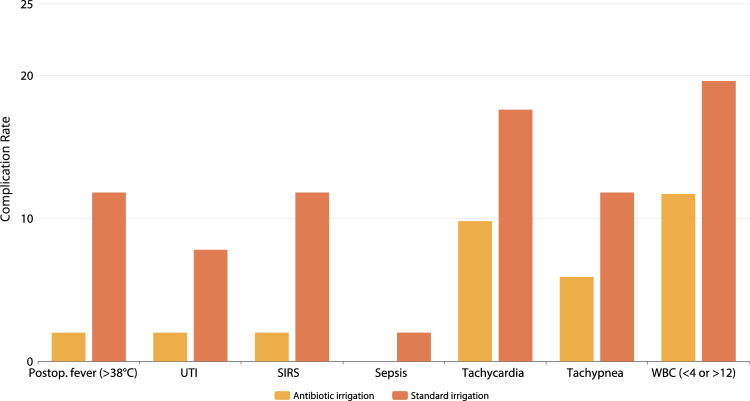
Postoperative fever, UTI, SIRS, sepsis, tachycardia, tachypnea, and abnormal WBC count in the antibiotic irrigation group and the standard irrigation group

The baseline information and the preoperative data of the patients are shown in Table 1. The patients comprised 67% males and 33% females, with a mean age of 47 ± 13 years. The intraoperative and postoperative data of the patients are shown in Table 2. Statistically significant differences were determined between the groups with respect to postoperative fever (*p* = 0.05), SIRS (*p* = 0.05), and postoperative length of stay in the hospital (*p* = 0.05). There was no significant difference between the groups with respect to the stone-free rate (*p* > 0.05). Only one patient had a positive blood culture. This patient who was diagnosed with sepsis also had a positive urine culture. As the production in the blood culture was determined to be of E.coli origin, the UTI diagnosis was made. The rates of postoperative fever, UTI, SIRS, sepsis, tachycardia, tachypnea, and abnormal WBC count of the antibiotic irrigation and standard irrigation groups are shown in Fig. 1.

**Table 1:** Demographic characteristics of the patients and location, volume, Hounsfield unit, and S-ReSC score of the kidney stones

	Total (*n* = 102)	Antibiotic irrigation (*n* = 51)	Standard irrigation (*n* = 51)	*p-*value
Age, years	47.4 ± 13.4	45.5 ± 12.5	49.2 ± 14.1	0.1
Sex				0.5
Female	33 (32.4)	15 (29.4)	18 (35.3)	
Male	69 (67.6)	36 (70.6)	33 (64.7)	
BMI (kg/m^2^)	29.3 ± 3.1	29.6 ± 3.8	28.6 ± 3.1	0.1
ASA score				0.1
1	23 (22.5)	9 (17.6)	14 (27.5)	
2	53 (52.0)	32 (62.7)	21 (41.2)	
3	26 (25.5)	10 (19.6)	16 (31.4)	
Stone location				0.4
Right	52 (51.0)	28 (54.9)	24 (47.1)	
Left	50 (49.0)	23 (45.1)	27 (52.9)	
Stone volume, mm^3^	986 (844–1016)	942 (884–996)	1008 (932–1064)	0.1
Stone density HU, peak	916 ± 252	928 ± 264	890 ± 238	0.4
S-ReSC score	4.5 ± 2.2	4.3 ± 2.2	4.6 ± 2.3	0.5

Data are shown as n (%) and mean ± SD or median (interquartile range)

*BMI* body mass index, *ASA* American Society of Anaesthesiologists, *HU* Hounsfield unit, *S-ReSC* Seoul National University Renal Stone Complexity

**Table 2:** Early postoperative complications, stone-free rate, and intraoperative and postoperative 1 month follow-up data

	Total (*n* = 102)	Antibiotic irrigation (*n* = 51)	Standard irrigation (*n* = 51)	*p-*value
Presence of fever (> 38 °C)	7 (6.9)	1 (2.0)	6 (11.8)	0.05*
UTI	5 (4.9)	1 (2.0)	4 (7.8)	0.1
SIRS	7 (6.9)	1 (2.0)	6 (11.8)	0.05*
Sepsis	1 (1.0)	–	1 (2.0)	
Tachycardia	14 (13.7)	5 (9.8)	9 (17.6)	0.2
Tachypnea	9 (8.8)	3 (5.9)	6 (11.8)	0.2
Abnormal WBC count (< 4 or > 12)	16 (15.6)	6 (11.7)	10 (19.6)	0.2
SFR	74 (72.5)	36 (70.6)	38 (74.5)	0.6
SICU admission	3 (2.9)	1 (2.0)	2 (4.0)	0.5
POD 1 creatinine difference	0.04 ± 0.19	0.02 ± 0.21	0.05 ± 0.17	0.3
Operation time, min	57.3 ± 17.4	56.2 ± 16.9	58.4 ± 18.0	0.5
Postoperative hospital duration, day	1.1 ± 0.5	1.0 ± 0.4	1.3 ± 0.6	0.05*
Hospitalization within 30 days of RIRS	10 (9.8)	3 (5.9)	7 (13.7)	0.1

Data are shown as *n* (%) and mean ± SD

*UTI* urinary tract infection, *SIRS* systemic inflammatory response syndrome, *WBC* white blood cell, *SFR* stone-free rate, *SICU* surgical intensive care unit, *POD* postoperative day, *RIRS* retrograde intrarenal surgery

(*) A statistically significant difference

With the exception of patients with postoperative fever, UTI, SIRS, sepsis, and SICU admission, all the other patients were discharged on postoperative day 1. Acute renal failure did not develop in any patient. No operation lasted longer than 90 min. There was no requirement for blood transfusion in any patient, and no severe complications such as ureteral perforation or avulsion were observed.

## Discussion

Together with the increasing prevalence of kidney stones, greater numbers of ureteroscopy procedures are being performed [7]. Ureteroscopy applied for large kidney stones currently has a small risk of major complications and good stone-free rates [8]. However, despite the decrease in major complications, an increase is predicted in infectious complications and mortality following ureteroscopy [9, 10].

In a systematic review by the EAU Section of Urolithiasis (EULIS), it was reported that the total complication rate was 7.9%, infectious complications were seen at 3.9%, and postoperative fever at 2.6% following ureteroscopy for stone disease [3]. Rates of postoperative fever have been reported as 8.9 and 22.8% in different groups [11, 12]. The mortality rate after ureteroscopy in renal stone disease has been reported as 0.8%, of which sepsis constituted 28% [10]. In light of these data, it is inevitable that the primary target must be to reduce infectious complications following ureteroscopy for renal stones.

Antibiotic treatment before RIRS has been accepted in all areas with respect to preventing infectious complications [3], but no proven method has been determined as yet which aims to reduce infectious complications by sterilizing the collecting system during lithotripsy in RIRS. A dynamic intrarenal pressure (IRP) profile is observed during RIRS, with IRP usually exceeding the expected levels, and there has been reported to be a relationship between high IRP and postoperative urosepsis [13]. Stone culture and renal pelvis urine culture have been proven to be better in the prediction of infectious complications compared to mid-flow urine culture [5, 6]. When it is considered that stone or renal pelvis culture is better than mid-flow culture in predicting infectious complications and urosepsis that can develop after exceeding expected IRP levels during RIRS, the use of an antibiotic solution that can provide sterilization during lithotripsy may be an ideal method to reduce infectious complications. Gentamicin has been proven to be a suitable antibiotic for safe long-term use in solutions for the treatment of gram-negative organisms, to reduce the incidence of colonization or infection, and for prosthesis rescue and intravesical instillation [14, 15].

Postoperative fever (> 38 °C) in the current study was consistent with literature at the rate of 11.8% in the standard irrigation group and was determined to be significantly lower at 2% in the antibiotic irrigation group. This showed that the infectious complication most frequently seen after RIRS was significantly reduced. Similarly, SIRS prevalence was consistent with the literature in the standard irrigation group and was determined at the low rate of 2% in the antibiotic irrigation group. Sepsis was seen in one patient in the standard irrigation group and in none of the antibiotic irrigation group. As was seen in this study, infectious complications may develop despite a negative urine culture. Therefore, this antibiotic irrigation protocol, which can be administered to all patients undergoing RIRS, becomes more important for the reduction of postoperative infectious complications. One of the most important points of the current study was the evaluation of factors such as operating time, stone size, localization of the stone within the kidney, age, gender, and ASA score, all of which can affect postoperative infectious complications [3], and no significant difference was determined between the groups. In addition, the requirement for preoperative negative urine culture in all the patients and the administration of antibiotic prophylaxis eliminated many factors that can affect complications. The postoperative length of stay in the hospital was significantly shorter in the antibiotic irrigation group. This was due to the need for clinical follow-up before discharge in patients with postoperative fever, SIRS, or sepsis. When the groups were evaluated with respect to operation success, there was no significant difference between the antibiotic irrigation group and the standard irrigation group, and a stone-free rate of 70% was obtained, which was consistent with the literature. From this, it was concluded that the antibiotic irrigation during the operation had no effect on laser efficacy or field of vision. Moreover, no adverse reaction or complication was seen to be directly related to antibiotic irrigation.

There were some limitations to this study, in which a new protocol was evaluated, primarily that stone composition was not examined. As stone fragments were not extracted, intraoperative stone samples could not be obtained. Infected stones such as struvite stones can increase complication rates [11]. A further limitation was that stone or renal pelvis cultures could not be taken intraoperatively. However, considering that there could be bias due to false negative results in the group where the antibiotic solution was used from the start of ureteroscopy, it was not planned to take pelvis or stone cultures intraoperatively. In addition, as this study was conducted in a single center, the results cannot be generalized to other centers, so there is a need for further multicenter studies. For this protocol to be able to be used as standard treatment, especially in patients at high risk of infectious complications, further, prospective, long-term studies with larger samples are required.

## Conclusions

A new protocol is presented in this study for sterilization of the renal collecting system using antibiotic irrigation during lithotripsy in RIRS. Due to the ease of application, it could become a part of standard RIRS. This antibiotic irrigation protocol is a promising method, which can be used safely and effectively to reduce infectious complications such as postoperative fever and SIRS without affecting the success of the surgical procedure.

## Data Availability

Data are not publicly available but can be made available by the corresponding author upon reasonable request.
